# Examining word association networks: A cross-country comparison of women’s perceptions of HPV testing and vaccination

**DOI:** 10.1371/journal.pone.0185669

**Published:** 2017-10-05

**Authors:** Bernd C. Schmid, Jamie Carlson, Günther A. Rezniczek, Jessica Wyllie, Kenneth Jaaback, Filip Vencovsky

**Affiliations:** 1 Department of Gynaecological Oncology, Royal Hospital for Women, Randwick, Australia; 2 Newcastle Business School, Faculty of Business and Law, University of Newcastle, Australia, Newcastle, Australia; 3 Department of Obstetrics and Gynecology, Ruhr-Universität Bochum (Marien Hospital Herne), Düngelstraße 33, Herne, Germany; 4 Department of Gynaecological Oncology, University of Newcastle, School of Medicine and Public Health, John Hunter Hospital, New Lambton Heights, Australia; 5 Faculty of Informatics and Statistics, University of Economics, Prague, Czech Republic; Duke University, UNITED STATES

## Abstract

In this study, we examined the perceptual associations women hold with regard to cervical cancer testing and vaccination across two countries, the U.S. and Australia. In a large-scale online survey, we presented participants with ‘trigger’ words, and asked them to state sequentially other words that came to mind. We used this data to construct detailed term co-occurrence network graphs, which we analyzed using basic topological ranking techniques. The results showed that women hold divergent perceptual associations regarding trigger words relating to cervical cancer screening tools, i.e. human papillomavirus (HPV) testing and vaccination, which indicate health knowledge deficiencies with non-HPV related associations emerging from the data. This result was found to be consistent across the country groups studied. Our findings are critical in optimizing consumer education and public service announcements to minimize misperceptions relating to HPV testing and vaccination in order to maximize adoption of cervical cancer prevention tools.

## Introduction

Growing evidence has demonstrated that the human papillomavirus (HPV) is the most common sexually transmitted infection (STI) responsible in a range of cervical, anogenital and oropharyngeal cancer cases. Specifically, 83% of all cervical cancer cases worldwide are attributable to the HPV infection and are therefore preventable through vaccination and screening tools [1]. In spite of the varying early cervical cancer detection programs (ECDP) that exist across the globe, cervical cancer is the fourth most widespread cancer affecting women worldwide, with an estimated 527,624 new cases and 265,672 deaths since 2012 [2, 3]. Although proven to be effective in decreasing the incidence and mortality rates of cervical cancer, cytology screening programs with a call and recall system [4] have begun to be replaced with HPV testing and vaccination as primary ECDP screening tools in several countries [5, 6].

In contrast to cervical cytology, new evidence and technology has illustrated that HPV testing provides a cost-effective and more sensitive approach in detecting high-degree lesions [7], and consequently improves early detection amongst women. However, empirical research has found that despite the benefits afforded by HPV vaccination and testing, women often hold negative psychosocial and socio-cultural associations towards cervical cancer and screening [5]. These negative associations may include beliefs that cervical cancer is HIV-related and due to poor vaginal hygiene, so than screening signifies an admission of infidelity, or that screening may affect fertility [8]. Consequently, these associations may function as a deterrent towards proactive health behaviors amongst women (i.e. HPV vaccination and testing).

Variation in cervical cancer cases has therefore been linked to the presence of adequate ECDP and other relevant resources within countries, as well as the population presence of the cervical HPV infection [8]. For example, approximately half of all OECD countries have organized screening and vaccination via population-based programs [9]. Hence, with the population of women aged 15 years and older exceeding 2.7 billion worldwide [4], the efficacy of ECDPs requires commitment from the public with response to vaccination messages and cervical cancer screening recommendations [6]. Therefore, it is critical that public attitudes and perceptions of HPV vaccination and new screening methods like HPV testing are captured and understood. Such an understanding will aid in the optimization of consumer education, public service announcements and branding strategies that help to facilitate participation in vaccination and screening by women.

The sensitive, personal and private nature surrounding public health concerns and, in this context, cervical cancer and screening, has often resulted in participants being unwilling to answer direct questions [10, 11]. Thus, research designs employed in these studies need to reflect and adjust to these complexities accordingly, in a manner sensitive to the research participants. Due to these reasons, survey research has become very popular in health research [12, 13, 14, 15]. Survey research facilitates fast and cost-effective data collection, particularly when paired with online collection methods. It also facilitates highly structured data collection, which is useful when efficiency and, intuitive and quickly actionable outcomes are the focus of the research [16, 17]. To increase the sophistication of survey research techniques and to gather the data necessary for detailed research into consumer associations, researchers have developed approaches combining survey tasks and network-based associative analyses [18, 19, 20]. Building on this work, we demonstrate the utility of novel data-driven approaches to public health research, and propose these as a means to learn more about women’s perceptions of ECDP screening tools.

Utilizing this data-driven approach, in this study we analyze the perceptual word associations women hold with regard to ECDP screening tools (i.e. cervical cancer testing and vaccination) across two countries, the United States and Australia. We examined these two countries as they use the ECDP screening tools in different ways, thus enabling us to see how informed women drawn from the general population are and to identify information gaps within each country. We undertook a semi-structured data-mining exercise, which enabled the construction of co-occurrence network graphs that were then analyzed using basic topological ranking techniques. To this end, we aimed to answer the following research questions:

1. What word associations surrounding HPV testing do women hold and are these associations consistent across similar country groups?
2. What word associations surrounding HPV vaccination do women hold and are these associations consistent across similar country groups?
3. What can the types of terms produced and the connections between them tell us about the usefulness of word-association research in the public health context?

### Early Cervical Cancer Detection Programs (ECDP) in Australia and United States

ECDP screening, which until recently, was based solely on cytology, in the form of the Pap smear, is currently shifting to rely on HPV testing procedures instead (accompanied by a HPV vaccination program) [5, 21]. For instance, the National Cervical Screening Program in Australia is set to introduce HPV testing in December 2017 [7], while countries such as the United States and Mexico have had HPV testing and co-testing (cytology and HPV testing) as their primary screening tools since 2008 [6,7]. Subsequently, clinical guidelines aligned to frequency and age of cervical cancer screening vary across countries and are reflective of the ECDP screening tool.

Currently in Australia, the Pap test is the primary ECDP screening tool, with clinical guidelines recommending women aged 18 to 69 undergo a Pap test every two years [7]. This varies significantly with HPV vaccine and testing, such that young women (9 to 13 years of age) have two doses of the HPV vaccine, and HPV testing is recommended every five years for women aged 25 to 74 [7, 22].

In the United States, these cervical cancer clinical guidelines differ, with women aged 21 to 65 years recommended to undergo a Pap test every three years [23]. Further, women aged 30 to 65 years seeking to extend the screening interval are able to do so through a preferred method of co-testing, which comprises both cytology and HPV testing and is performed every five years [23]. However, it is necessary to note that average at-risk women aged 25 to 65 years have the ability to use the HPV test as their primary screening tool [7]

Based on the above discussion, Australia and the United States share similar ECDPs. However, a core difference between these two countries lies in their use of HPV vaccination and testing. Therefore, we aim to generate understanding of the perceptual associations that arise from women’s thinking about ECDP screening tools (i.e. Pap test, HPV testing and HPV vaccination) across these two countries. A key aspect of our work is that co-occurrence network graphs will enable greater understanding surrounding cervical cancer screening and, ultimately, work towards the optimization of consumer education and public service announcements.

## Methods

### Data collection

Using the consumer database from a reputable marketing research firm (SurveyMonkey), a large-scale online survey was conducted December 18–21, 2015. Participants were randomly selected from SurveyMonkey’s U.S. and Australian databases, using this study’s pre-defined selection criteria of women aged 18 to 64 years. An email invitation was sent to potential participants outlining the purpose of the study, giving instructions to complete the survey and including the link to the online survey. Implied consent to the study was provided through participants’ registration with SurveyMonkey, as well as the anonymous completion of this study’s survey. Participants who completed the survey were compensated via non-monetary incentives including donations to their preferred charity, and were given entries into a draw to win sweepstakes [24]. Further, consistent with institutional review board policies, ethics approval was not required.

A total sample of 1473 (68%) was achieved with 704 from the U.S. and 769 from Australia. The total number of incomplete responses was 697 accounting for 32% of the sample, with 346 of these, participants from the U.S. sample and 351 participants from the Australian sample. SurveyMonkey also provided basic demographic information from participants, such as age and household income brackets, which is summarized in Table 1.

**Table 1:** Overview of survey participants.

Sample Characteristics	Australia	U.S.A.	Total
***Sample Size***	769 (52%)	704 (48%)	1473 (100%)
***Age group***			
18 to 29	190 (25%)	161 (23%)	351 (24%)
30 to 44	270 (35%)	225 (32%)	495 (34%)
45 to 59	270 (35%)	278 (39%)	548 (37%)
60+	39 (5%)	40 (6%)	79 (5%)
***Household income***^*1*^			
$0 to $9,999	88 (11%)	112 (16%)	200 (14%)
$10,000 to $24,999	123 (16%)	156 (22%)	279 (19%)
$25,000 to $49,999	121 (16%)	122 (17%)	243 (16%)
$50,000 to $74,999	155 (20%)	88 (13%)	243 (16%)
$75,000 to $99,999	35 (5%)	63 (9%)	98 (7%)
$100,000 to $124,999	51 (7%)	71 (10%)	122 (8%)
Not Provided	196 (25%)	92 (13%)	288 (20%)
***Diagnosed with a Sexual Transmitted Infection (STI)***			
Yes	239 (31%)	254 (36%)	493 (34%)
No	530 (69%)	450 (64%)	980 (67%)

Values are n (%).

^1^ Household income groups were defined by SurveyMonkey’s demographic information. Dollar amounts for the Australian sample are in AU and dollar amounts for the U.S. sample are in USD

In the survey, we randomly presented participants with several trigger words to which they were asked to provide, in sequential order, the first three words (i.e. response words) that came to mind. The trigger words shown to participants comprised “cervical cancer”, “cervical cancer testing” and “cervical cancer vaccination” in succession. We divided the network analysis of participants’ response words (and subsequent presentation of results) into “vaccination” (trigger words “HPV vaccination” and “cervical cancer vaccination”) and “testing” (trigger words “HPV (human papillomavirus) test” and “pap smear”), and then further divided the responses by country groups (U.S. and Australia). For example, an Australian participant shown the trigger word ‘HPV (Human papillomavirus) test’ provided the response words of “cervix”, “cancer” and “virus”.

### Analysis

We used the data from the surveys to construct detailed, weighted term co-occurrence network graphs. Co-occurrence is a fundamentally simple concept, with relevance in “hard-science” applications [25] and social science applications such as analyzing textual co-occurrence patterns [26]. In this study, data was processed (including computing co-occurrence) using KNIME [27]. Network analysis was performed using Gephi [28] and Cytoscape [29].

Co-occurrence between response words was computed by taking the n-gram (i.e. set of adjacent words) co-occurrence statistic data [30] that participants typed into separated fields in response to the trigger words. Whatever participants entered into the three separate fields provided for each trigger word was then converted into three separate nodes. Subsequently, calculation of these co-occurrences between fields was conducted. The only pre-processing applied was case conversion (i.e. conversion to lower case) as we were only interested in ranking exactly matched n-grams in this study.

We also removed n-grams related to the terms “unknown” and “n/a” provided by participants as we took these to denote a non-response. We included all other n-grams. Edges were thus created connecting n-grams provided by the same unique participant, in response to the same trigger word. The co-occurrence data for each participant was then merged into separate network graphs according to each trigger word. When the participant-level data was combined, the nodes representing identical entries (in response to trigger words) were merged. Identical co-occurrence pairings (edges) were also merged. This approach enabled the most salient n-grams (i.e. nodes) to emerge as naturally as possible.

### Topology

Having generated networks from the word co-occurrence data collected from participants, we then analyzed the topological properties of the resulting networks. This involved examining the sub-structures of the networks (i.e. groupings of nodes and patterns in connections between nodes), as well as ranking nodes using some basic topological measures [31]. Specifically, we computed: degree centrality (the number of connections for each node), weighted degree (number of connections adjusted for edge weight) [32] and eigenvector centrality (weighted centrality, i.e. nodes with important connections get higher ranks) [33]. These measures were computed using the full networks (see Table 2) but, for clarity of presentation, we visualize and display rankings for nodes with degree centrality >10 only.

**Table 2:** Testing node rankings.

Testing															
U.S.								Australia							
U.S. HPV test	Deg	W Deg	EvC	U.S. Pap	Deg	W Deg	EvC	Aus HPV test	Deg	W Deg	EvC	Aus Pap	Deg	W Deg	EvC
cancer	96	175	1	uncomfortable	132	304	1	cancer	97	200	1	uncomfortable	154	446	1
necessary	76	111	0.641	necessary	104	260	0.837	doctor	72	122	0.862	necessary	116	330	0.871
std	69	103	0.675	yearly	80	146	0.7	necessary	71	116	0.642	cancer	69	158	0.645
new	66	86	0.612	test	72	135	0.634	new	63	83	0.627	doctor	65	152	0.608
test	61	83	0.637	cancer	66	126	0.639	unsure	58	78	0.512	test	63	128	0.542
good	49	74	0.45	painful	65	124	0.575	uncomfortable	57	101	0.681	invasive	56	108	0.552
disease	48	68	0.48	doctor	51	100	0.592	important	51	69	0.519	awkward	56	120	0.553
prevention	47	66	0.516	annual	50	94	0.537	good	48	67	0.498	vagina	54	94	0.489
scary	46	56	0.448	routine	45	56	0.455	test	48	93	0.537	embarrassing	53	124	0.504
virus	46	98	0.461	prevention	44	78	0.497	prevention	45	76	0.614	important	49	82	0.471
important	45	72	0.531	cold	44	78	0.439	virus	41	70	0.457	painful	47	95	0.531
sex	45	63	0.488	pain	40	51	0.42	warts	39	65	0.496	pain	42	60	0.411
doctor	41	57	0.458	vagina	40	56	0.379	std	39	60	0.506	prevention	39	70	0.452
safe	39	48	0.417	needed	39	62	0.468	smear	35	48	0.515	discomfort	38	48	0.361
preventative	34	40	0.442	important	35	62	0.42	disease	34	49	0.424	speculum	38	62	0.426
exam	34	44	0.415	health	34	44	0.379	preventative	34	45	0.436	regular	33	56	0.403
helpful	34	48	0.375	invasive	34	46	0.431	women	32	38	0.482	cervix	32	58	0.378
screening	31	39	0.464	exam	33	42	0.392	pap smear	32	62	0.429	safe	32	40	0.354
easy	30	38	0.304	safe	31	36	0.31	herpes	32	43	0.349	scary	28	34	0.38
needed	29	38	0.42	speculum	30	46	0.363	invasive	30	43	0.409	needed	28	36	0.3
warts	28	36	0.262	good	29	36	0.296	safe	28	38	0.381	annoying	27	38	0.281
women	27	32	0.386	swab	28	30	0.286	detection	27	38	0.388	cold	26	36	0.328
yearly	25	30	0.313	easy	26	28	0.209	scary	26	33	0.276	yuck	26	31	0.305
health	24	28	0.298	yuck	26	44	0.314	needed	25	30	0.319	ouch	24	26	0.246
smart	23	25	0.254	vaginal	26	38	0.354	sex	25	36	0.366	quick	23	34	0.193
painful	22	23	0.271	annoying	25	36	0.304	vagina	24	42	0.387	old	23	34	0.245
young	22	23	0.293	ouch	25	28	0.31	hiv	23	28	0.229	essential	23	32	0.254
teens	21	24	0.249	women	24	28	0.269	easy	22	32	0.243	health	23	36	0.298
blood	21	26	0.31	stirrups	24	32	0.283	essential	21	22	0.22	yearly	23	26	0.227
no	21	30	0.08	preventative	24	38	0.377	discomfort	20	24	0.245	women	21	38	0.309
uncomfortable	21	27	0.264	gross	23	38	0.294	useful	20	22	0.256	examination	21	28	0.264
unsure	21	25	0.165	awkward	23	44	0.346	cervix	20	32	0.34	embarrassing	21	26	0.243
detection	21	26	0.271	screening	23	38	0.374	screening	20	28	0.329	good	21	26	0.264
cervix	20	28	0.322	old	21	22	0.174	medical	20	23	0.334	annual	21	22	0.254
pap	19	27	0.291	embarrassing	21	30	0.306	accurate	19	22	0.19	routine	21	26	0.166
shot	17	21	0.202	helpful	20	28	0.306	better	19	20	0.198	preventative	20	28	0.254
vaccine	17	18	0.239	normal	20	24	0.185	what	18	19	0.13	detection	19	28	0.261
pap smear	17	18	0.214	smear	19	44	0.201	cervical	18	30	0.244	cells	18	24	0.212
aids	17	22	0.188	gynecologist	18	26	0.27	prevent	18	20	0.258	unpleasant	18	32	0.248
what	17	19	0.091	no	18	20	0.121	vaccine	18	26	0.212	vaginal	18	22	0.257
expensive	16	16	0.076	detection	18	24	0.214	reliable	18	18	0.231	yuk	17	18	0.148
youth	16	18	0.169	gyno	16	16	0.236	helpful	18	27	0.265	intrusive	16	24	0.203
why	16	18	0.116	hurt	16	16	0.132	health	17	18	0.252	female	16	18	0.236
accurate	16	18	0.207	scary	16	20	0.156	cervical cancer	17	24	0.272	hpv	15	16	0.159
unfamiliar	15	15	0.128	pap	16	40	0.162	pain	16	18	0.132	smear	15	30	0.153
early	15	18	0.187	cervix	15	24	0.231	check	16	20	0.293	easy	14	22	0.086
useful	15	19	0.179	unpleasant	15	22	0.198	pap	15	22	0.251	no	14	21	0.064
sick	15	16	0.122	ugh	15	18	0.178	results	15	16	0.211	time	14	14	0.081
informative	15	16	0.181	testing	14	14	0.173	yuck	14	16	0.218	gross	13	16	0.188
hiv	15	20	0.128	healthy	14	22	0.233	great	14	14	0.102	useful	13	16	0.185
contagious	15	18	0.186	discomfort	14	14	0.167	human	14	29	0.237	check	13	16	0.16
need	14	16	0.086	check up	13	13	0.088	awkward	14	20	0.187	horrible	13	16	0.17
preventable	14	14	0.213	hurts	13	16	0.238	cells	14	16	0.201	avoid	12	14	0.166
effective	14	18	0.214	dread	13	16	0.159	sti	14	16	0.246	results	12	14	0.107
speculum	14	14	0.159	quick	13	20	0.184	female	14	17	0.222	hurts	12	14	0.197
dirty	14	18	0.167	fast	12	12	0.115	life saving	14	14	0.174	woman	11	12	0.135
gross	14	14	0.118	nervous	12	14	0.151	blood	14	16	0.154	doctors	11	12	0.144
better	14	18	0.147	preventive	12	16	0.174	blood test	13	14	0.154	cervical	11	22	0.134
knowledge	14	16	0.183	required	12	12	0.172	how	13	15	0.067	2 years	11	12	0.167
cervical	13	18	0.206	hate	12	12	0.195	vaccination	13	15	0.202	required	11	14	0.111
cervical cancer	13	14	0.132	female	11	14	0.163	same	13	16	0.118	cervical cancer	11	12	0.195
pain	12	12	0.085	cervical	11	14	0.126	painful	12	20	0.166	helpful	11	12	0.164
safety	12	14	0.134	same	11	12	0.124	examination	12	13	0.166	pap	11	21	0.079
routine	12	12	0.102	informative	11	14	0.178	needle	12	16	0.155	screening	11	12	0.153
swab	12	12	0.116	woman	11	12	0.212	quick	11	14	0.108	necessary	10	12	0.117
preventive	12	12	0.132	yucky	11	12	0.163	yes	11	16	0.084	embarrassing	10	16	0.179
results	12	12	0.206	embarrassing	11	16	0.118	early	11	16	0.163	reliable	10	12	0.094
annual	12	12	0.155	check	10	10	0.091	embarrassing	11	20	0.166	precaution	10	12	0.165
ok	11	12	0.045	smart	10	12	0.121	protection	11	14	0.186	embarrassment	10	12	0.129
smear	11	14	0.113	reliable	10	12	0.152	no	11	22	0.04	inconvenient	10	12	0.173
human	11	42	0.134	standard	10	10	0.157	ouch	10	10	0.145	scrape	10	12	0.15

### Visualization

Visualization was performed using Gephi [28], whereby node size corresponds with degree centrality, edge size corresponds with edge weight (i.e. the number of paired occurrences between nodes) and rank tables are ordered by degree centrality.

## Results

In the following sections, the network properties and structures of each of the developed ‘trigger word’ network graphs are discussed. The results show that the HPV and Pap smear testing networks illustrated similarities with the salient n-grams (i.e. “necessary”) that arose, whilst negative n-grams (i.e. “uncomfortable”) were most apparent in the Pap smear testing networks. Furthermore, upon visual inspection of the HPV and cervical cancer vaccination networks across both country groups, the preventative and beneficial nature of the trigger word “vaccination” was exhibited through the identified n-grams.

### HPV and Pap smear testing networks

**Fig 1:**
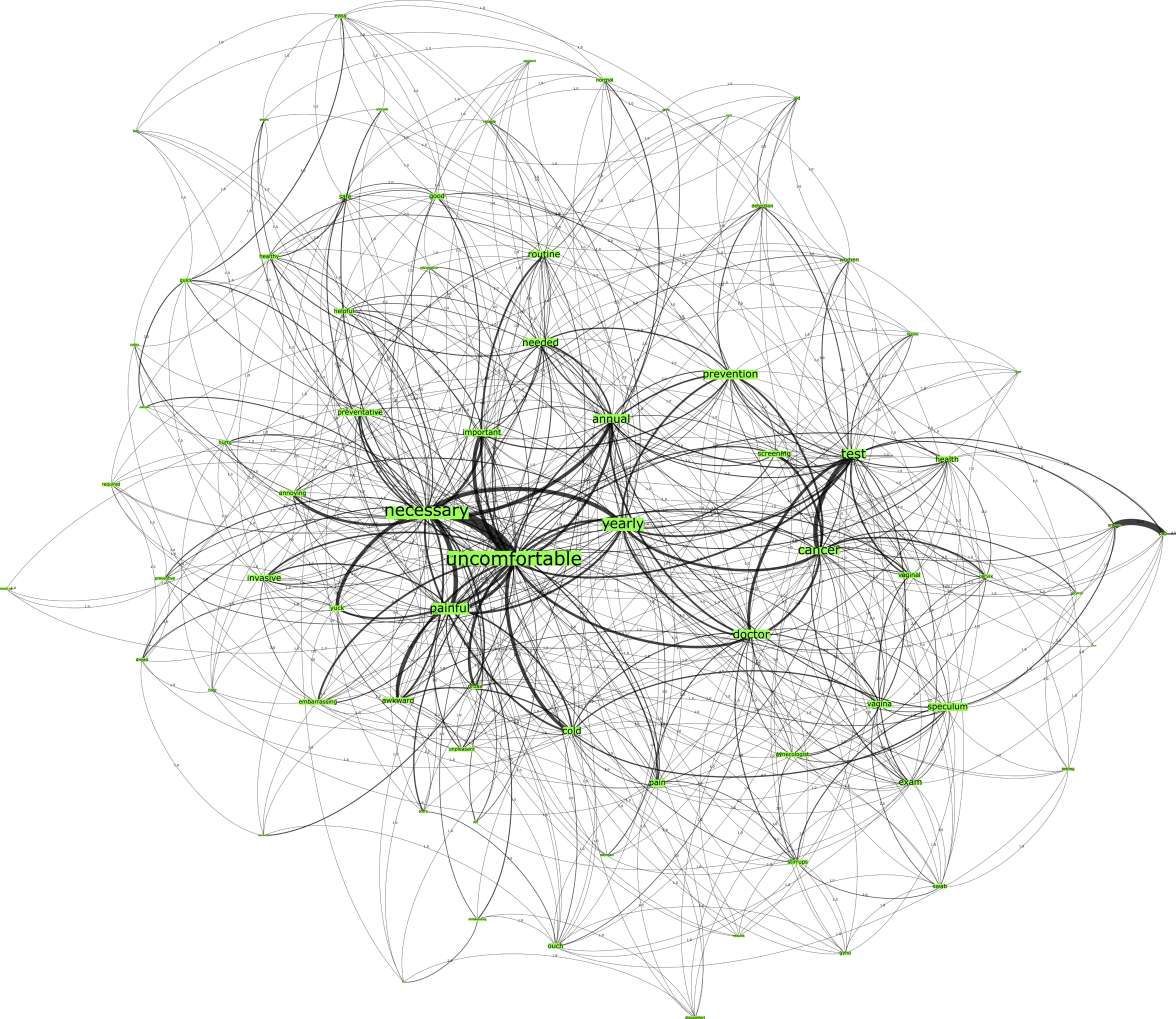
Network A. U.S. HPV Test network visualization.

Network A, seeded from the trigger word “HPV Testing” for the U.S. country group comprised, 794 nodes connected by 1974 edges with an average degree of 4.904 (Fig 1). This network graph partitions into four modules, with a modularity score of 0.334: one core community related to the n-gram “cancer”, two major communities related to the n-gram “necessary” and “prevention” and one disparate community. In line with the degree and eigenvector centrality measures specified in Table 2 for the U.S. country group, the top-ranked n-grams that are most embedded in the network are “cancer”, “necessary” and “std (sexually transmitted disease)”.

**Fig 2:**
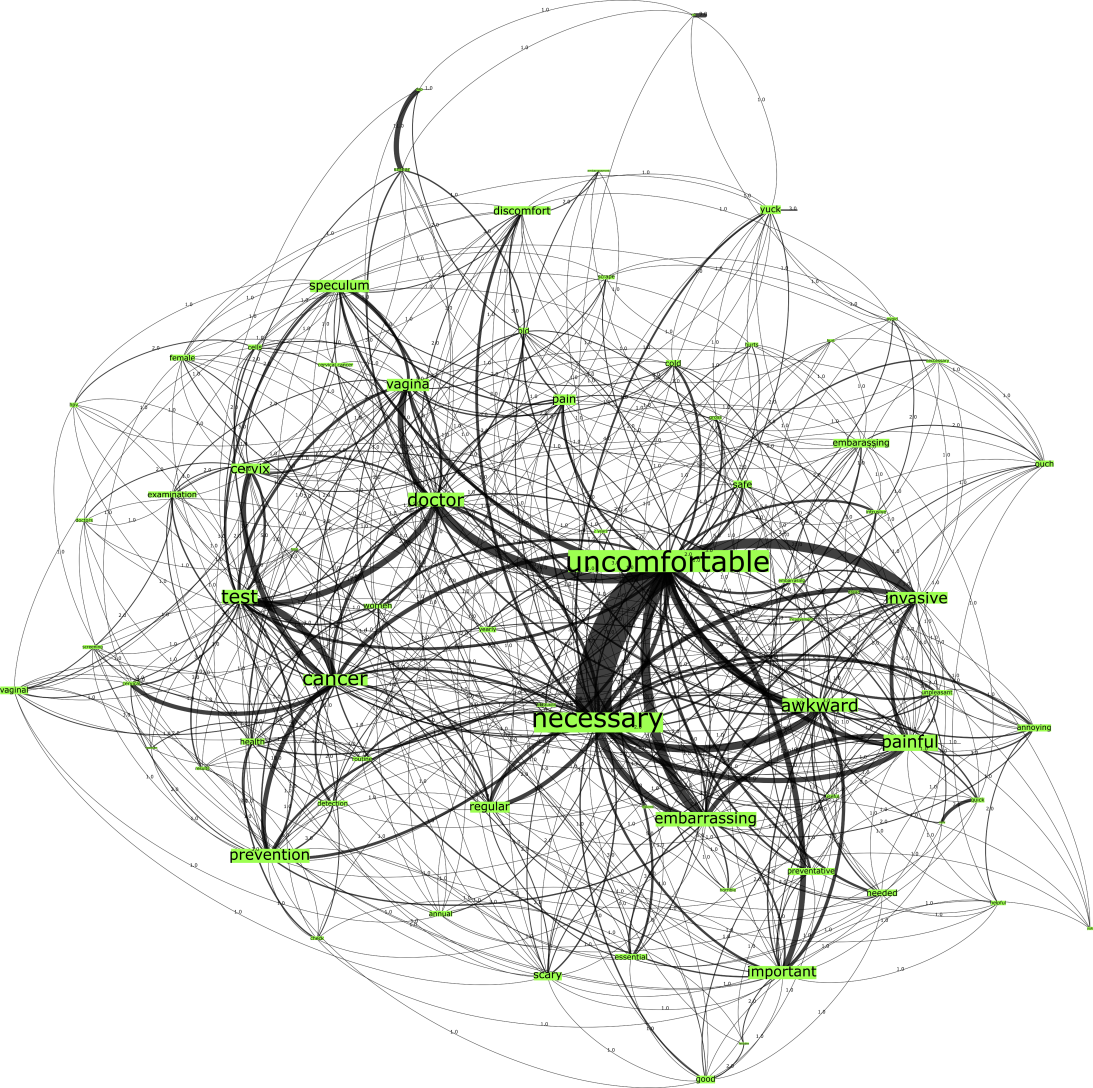
Network B. U.S. Pap smear test network visualization.

In reference to Network B, which was seeded from the trigger word “Pap smear testing”, the U.S. country group is comprised of 667 nodes connected by 1790 edges with an average degree of 5.367 (Fig 2). This network graph also partitions into four modules, with a modularity score of 0.25 and comprising one core community related to the n-gram “uncomfortable”, one major community related to the n-gram “test”, a small community related to the n-gram “annual” and a disparate community. Furthermore, as shown in Table 2, the top-ranked n-grams most embedded in this network were “uncomfortable”, “necessary” and “yearly”. Although studies have shown that HPV heightens the risk of cervical cancer in women [34], the term “std” does not feature within the top 25 nodes in the Pap smear test network graph. Overall, these findings demonstrate that HPV testing is perceived as being less invasive and more favorable than the Pap smear test amongst the U.S. female participants.

**Fig 3:**
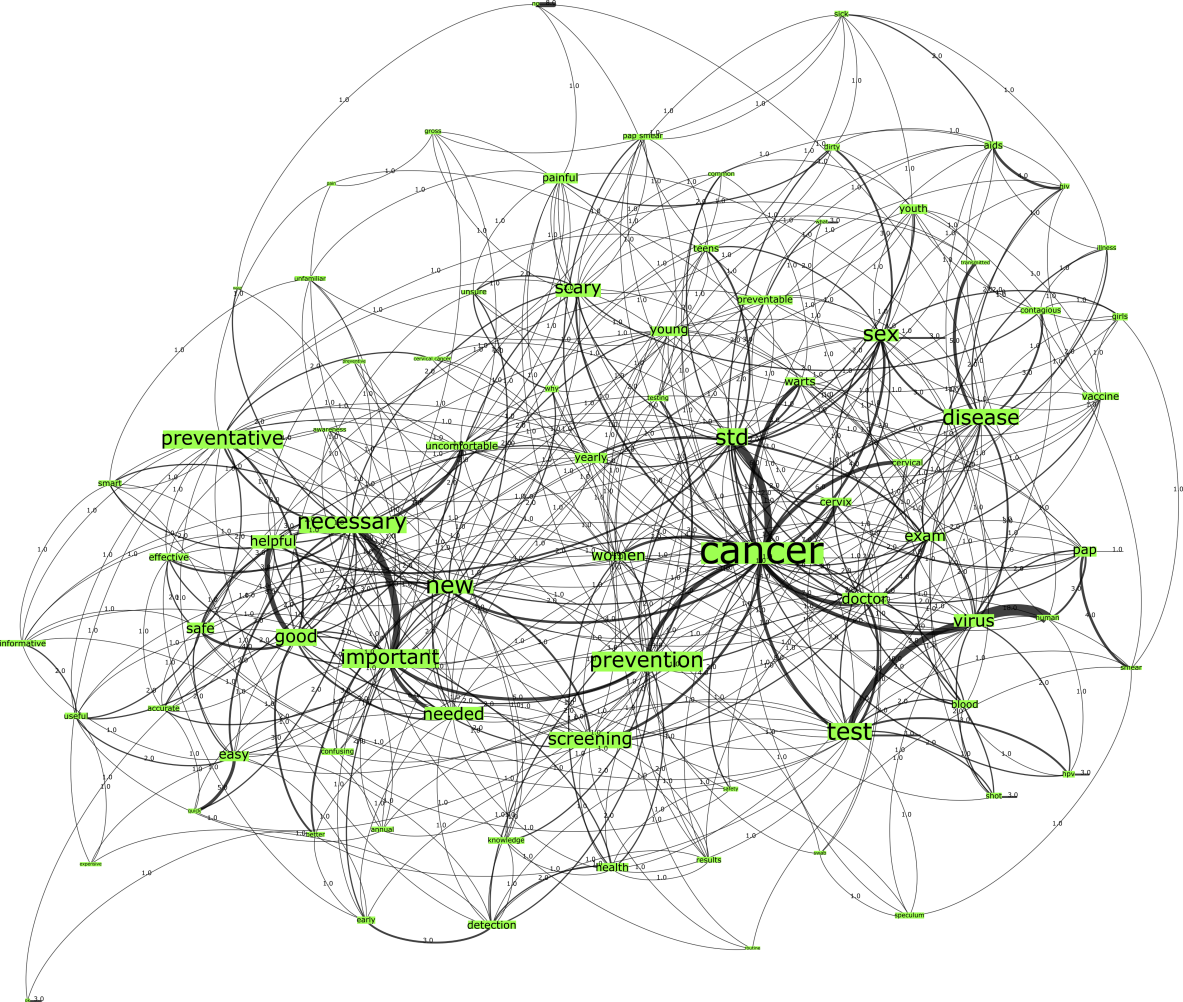
Network C. Australia HPV test network visualization.

In comparison to the U.S. country networks, the findings from the Australian network groups demonstrate similar n-grams. Specifically, Network C, seeded from the trigger words “HPV testing”, comprised 718 nodes connected by 1703 edges with an average degree of 5.136 (Fig 3). This network graph partitioned into three modules, with a modularity score of 0.316. As specified in Table 2, the network included one core community related to the n-gram “cancer” and two major communities related to the n-grams “necessary” and “good” respectively.

**Fig 4:**
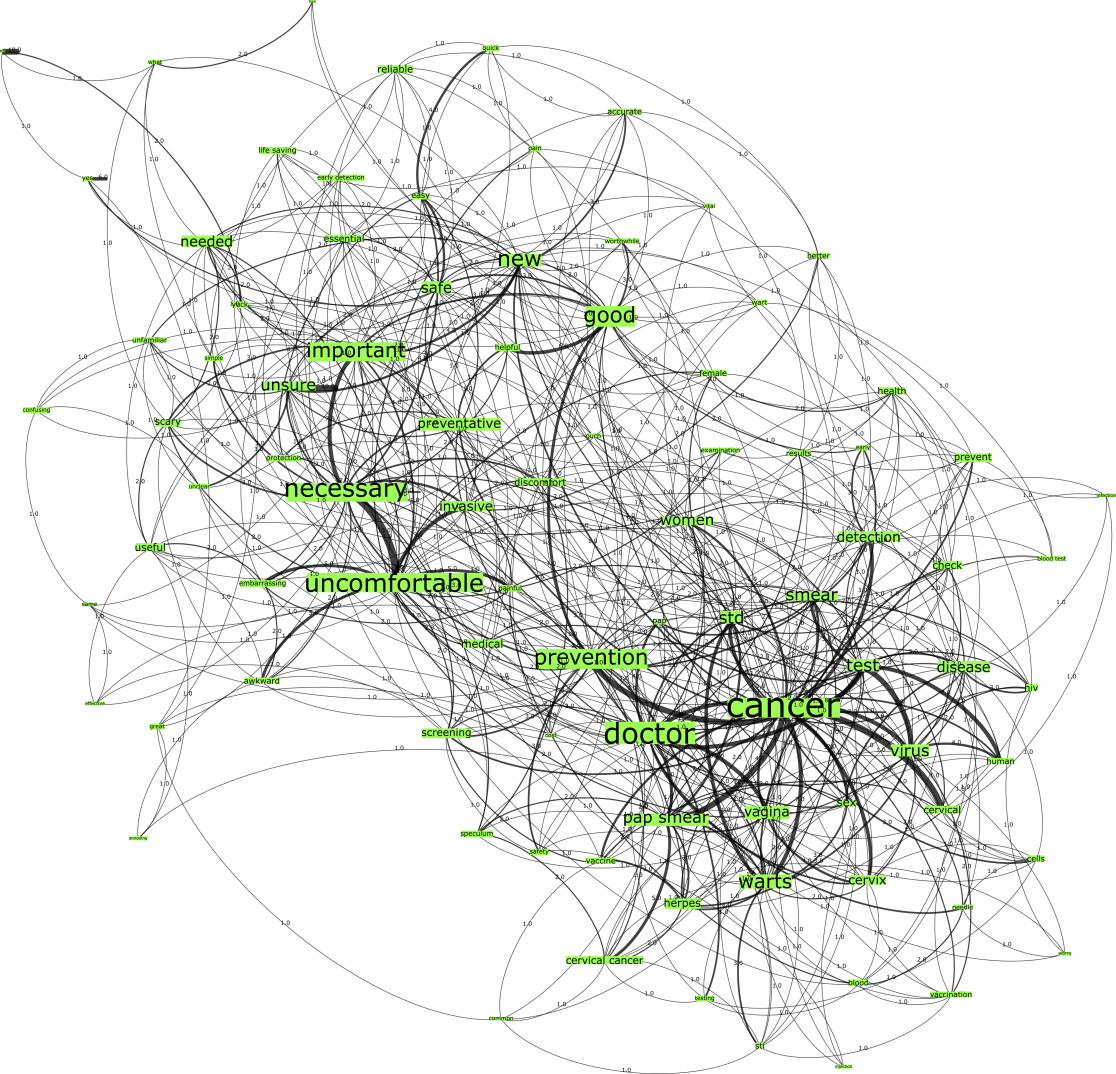
Network D. Australia pap network graph.

Network D, seeded from the trigger word “Pap smear testing” within the Australian sample, comprised 625 nodes connected by 1703 edges with an average degree of 5.45 (Fig 4). This network graph partitioned into four modules, with a modularity score of 0.242. It included one core community related to the n-gram “uncomfortable”, one major community related to the n-gram “cancer”, one small community related to the n-gram “discomfort” and finally one disparate community. Across both networks, C and D, the three most embedded n-grams within these networks comprised “cancer”, “uncomfortable” and “necessary”. However, the top-ranked n-grams of these network graphs were different, such that degree and eigenvector centrality showed that “cancer” was the top-ranked n-gram of Network C, whilst “uncomfortable” was the top-ranked n-gram in Network D. This result indicates’ that Australian women are more aware than women from the U.S. that the testing process of retrieving the small sample of cells from the surface of the cervix is actually the same for both, the HPV test and the Pap smear test.

Conversely, the n-grams from the HPV test network graphs (see Figs 1 and 3) across both country groups have demonstrated more positive associations than those of the Pap smear network graphs (see Figs 2 and 4). There is greater correlation evidenced between the n-grams “prevention”, “detection”, “screening” to the terms “cancer” and “doctor” in these graphs. Finally, the Australian network graphs (see Figs 3 and 4) highlighted that participants identified the timeframe in which testing is performed to detect cervical cell changes via the HPV test or Pap smear test with n-grams “yearly” and “annual”, which is surprising given the 2-year screening interval in Australia. This perception may heighten the perceived burden for women in undertaking this preventative behavior.

### HPV and cervical cancer vaccination networks

**Fig 5:**
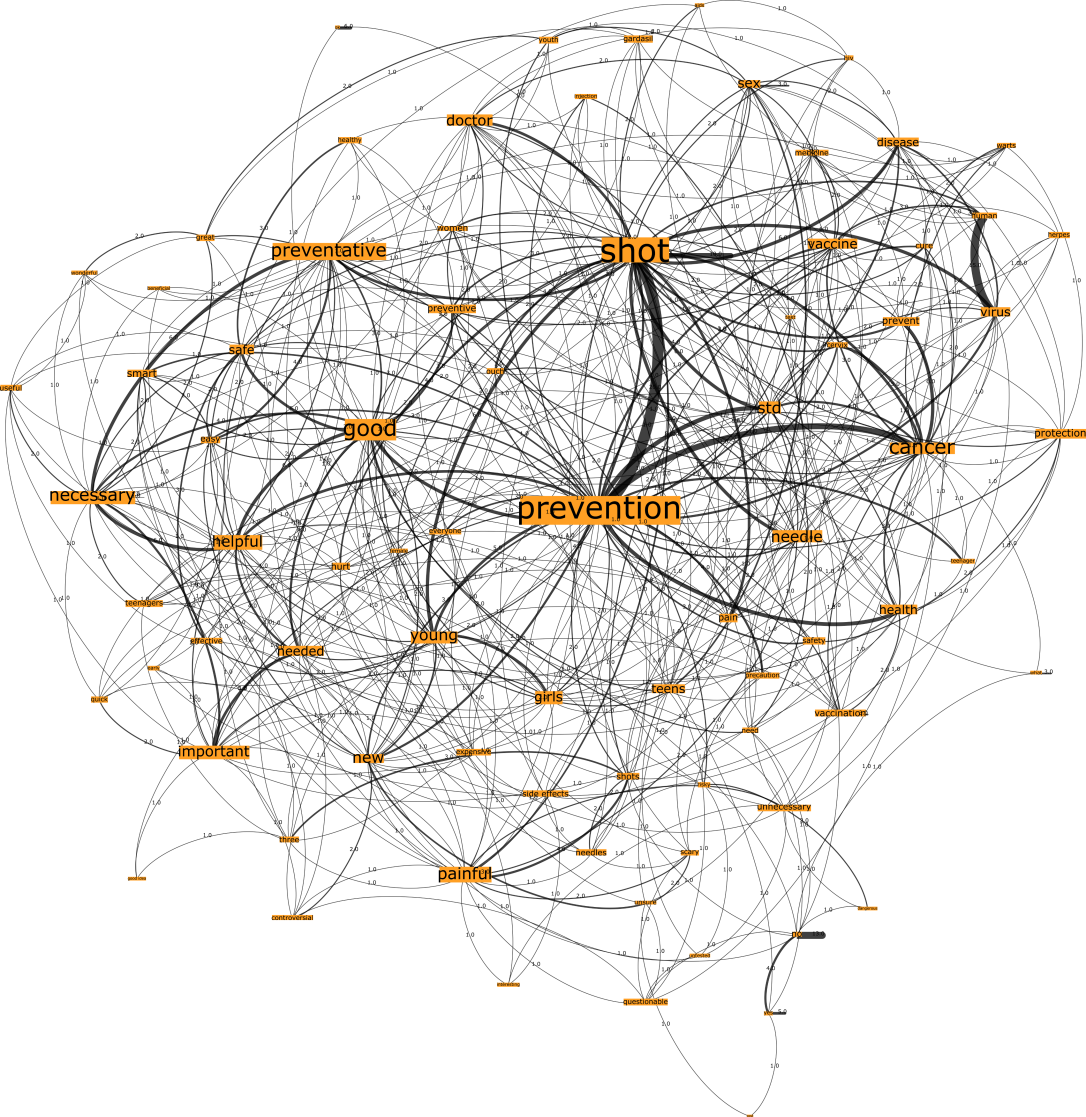
Network E. U.S. HPV vaccination network visualization.

**Fig 6:**
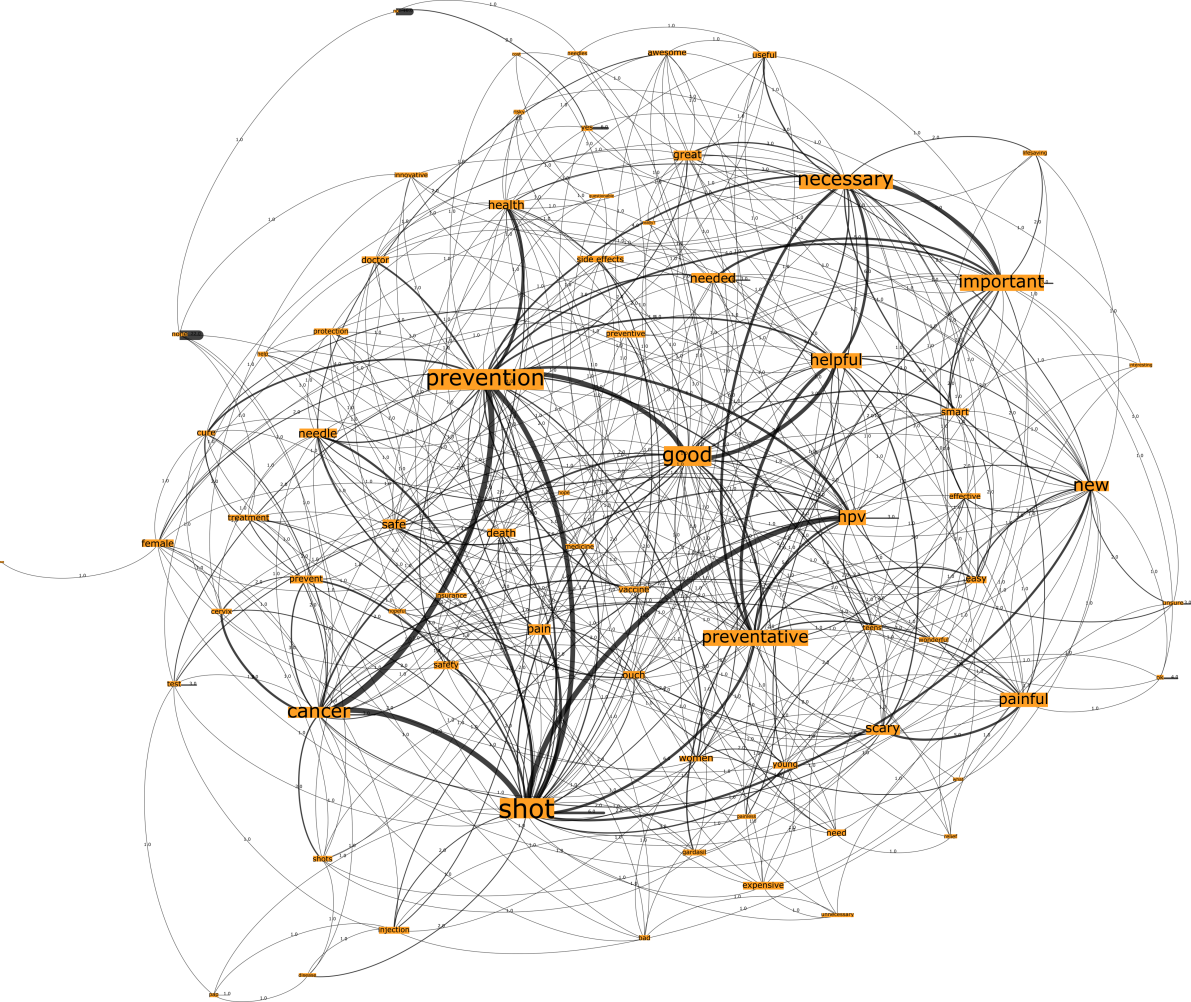
Network F. U.S. cervical vaccination network visualization.

Within the U.S. sample, Network E, seeded from the trigger words “HPV vaccination”, comprised 833 nodes connected by 1972 edges with an average degree of 4.735 (Fig 5). This network graph partitions into four communities, with a modularity score of 0.276. The communities included one core community related to the n-grams “prevention” and “shot”, one major community related to the n-gram “good”, and one small community related to the n-grams “young” and “painful”. Analysis of Network F, seeded from the trigger words “cervical cancer vaccination”, showed that the network comprised 828 nodes that were connected by 1957 edges with an average degree of 4.727 (Fig 6). Network F partitions into three modules with a modularity score of 0.226, with one core community related to the n-gram “shot”, a major community related to the n-grams “necessary” and “good”, and, finally, a small community related to the n-gram “prevention”. As shown in Table 3, the three most embedded n-grams and their ranks, across both Network E and F for the U.S. country group, were identical (“shot”, “prevention” and “good”).

**Table 3:** Vaccination node rankings.

Vaccination															
U.S.								Australia							
U.S. HPV Vac	Deg	W Deg	EvC	U.S. C Vac	Deg	W Deg	EvC	AUS HPV Vac	Deg	W Deg	EvC	AUS C Vac	Deg	W Deg	EvC
shot	122	224	1	shot	103	185	1	prevention	111	210	0.96	prevention	119	246	0.992
prevention	100	204	0.918	prevention	90	166	0.866	needle	108	215	1	needle	112	223	1
good	69	102	0.692	good	80	119	0.796	necessary	80	126	0.737	good	83	139	0.815
new	60	72	0.513	new	69	83	0.614	good	73	117	0.657	necessary	81	129	0.886
necessary	56	80	0.535	cancer	69	137	0.663	cancer	67	132	0.762	cancer	60	141	0.642
cancer	55	90	0.544	necessary	69	103	0.701	easy	56	90	0.554	preventative	55	76	0.572
important	52	64	0.464	helpful	61	93	0.692	preventative	53	74	0.541	important	55	77	0.677
preventative	51	70	0.472	important	57	81	0.574	injection	52	76	0.506	easy	54	92	0.552
painful	46	55	0.467	hpv	56	94	0.599	unsure	47	56	0.441	hpv	54	83	0.569
needed	44	58	0.431	preventative	48	80	0.583	safe	46	70	0.458	great	46	56	0.491
needle	43	54	0.422	painful	45	55	0.548	important	46	59	0.537	safe	45	71	0.549
helpful	42	63	0.415	scary	41	51	0.492	virus	45	86	0.461	injection	45	70	0.462
young	41	60	0.472	needed	35	45	0.473	doctor	44	69	0.538	doctor	39	57	0.562
safe	40	54	0.417	needle	33	44	0.422	new	42	55	0.408	women	33	50	0.433
std	39	57	0.419	pain	33	42	0.396	protection	40	50	0.461	protection	32	42	0.448
virus	36	70	0.362	safe	32	38	0.448	needles	37	46	0.348	helpful	32	42	0.396
teens	31	36	0.378	death	32	36	0.376	great	32	34	0.301	pain	32	44	0.314
doctor	30	38	0.404	health	31	40	0.443	painful	31	41	0.297	painful	31	34	0.252
no	30	44	0.165	great	31	35	0.357	health	29	43	0.373	health	30	46	0.359
health	28	38	0.373	easy	31	40	0.352	young	27	36	0.345	unsure	30	32	0.195
disease	28	42	0.323	women	30	36	0.39	teenagers	27	33	0.348	new	30	41	0.347
vaccine	28	40	0.34	doctor	24	28	0.314	effective	25	32	0.345	needles	29	36	0.361
protection	27	30	0.289	no	24	40	0.056	std	25	32	0.354	needed	28	36	0.321
easy	25	34	0.258	expensive	24	24	0.231	vaccine	24	36	0.317	girls	28	38	0.471
girls	24	32	0.441	need	21	22	0.266	girls	24	36	0.306	young	27	34	0.411
unnecessary	24	26	0.194	unsure	21	25	0.139	pain	22	28	0.268	essential	25	30	0.345
sex	24	33	0.366	prevent	20	26	0.256	warts	21	28	0.277	vaccine	25	38	0.311
preventive	23	30	0.245	side effects	19	20	0.255	uncomfortable	21	25	0.275	prevent	24	32	0.329
smart	23	28	0.35	young	19	24	0.321	what	21	24	0.192	ouch	23	30	0.32
prevent	22	32	0.24	ouch	19	25	0.339	useful	21	23	0.272	effective	22	28	0.319
vaccination	22	29	0.255	treatment	19	24	0.243	test	21	30	0.275	lifesaving	20	25	0.345
scary	20	22	0.179	cervix	19	26	0.2	essential	20	22	0.163	excellent	20	24	0.19
shots	20	28	0.221	smart	19	28	0.357	ouch	20	24	0.252	fantastic	19	20	0.202
human	20	46	0.179	protection	18	18	0.243	prevent	20	27	0.321	cure	18	22	0.263
dangerous	19	20	0.044	cure	18	24	0.222	quick	19	28	0.247	awesome	18	22	0.202
effective	19	22	0.211	unnecessary	18	18	0.097	school	19	26	0.288	free	18	22	0.275
needles	19	20	0.204	shots	18	20	0.17	helpful	18	22	0.265	scary	18	21	0.15
ouch	18	22	0.216	preventive	18	20	0.259	sex	18	22	0.259	no	18	31	0.119
expensive	18	18	0.239	risky	17	18	0.113	better	18	20	0.196	safety	16	22	0.259
side effects	18	19	0.214	safety	17	20	0.249	women	18	26	0.286	life	16	18	0.2
three	18	20	0.215	effective	17	20	0.282	cervical cancer	18	18	0.205	school	16	18	0.248
pain	17	22	0.253	injection	17	20	0.228	safety	18	19	0.207	breakthrough	16	18	0.237
great	17	21	0.151	what	17	18	0.118	simple	17	20	0.214	teenagers	16	21	0.246
quick	17	20	0.148	test	17	21	0.231	free	17	20	0.238	cervix	15	28	0.279
teenagers	16	18	0.227	female	17	18	0.289	disease	16	24	0.215	vagina	15	18	0.273
women	16	18	0.256	bad	16	16	0.151	herpes	16	20	0.232	yes	15	19	0.161
unsure	16	19	0.113	vaccine	16	22	0.297	cervical	16	26	0.291	useful	14	17	0.228
useful	15	16	0.169	awesome	15	16	0.288	no	16	23	0.094	female	14	18	0.236
cure	15	20	0.219	ok	15	18	0.167	medical	15	17	0.192	innovative	14	14	0.195
safety	15	18	0.181	insurance	15	16	0.23	needed	15	18	0.13	death	14	16	0.171
gardasil	14	14	0.175	gardasil	14	16	0.193	yes	14	15	0.095	compulsory	14	14	0.207
cervix	14	22	0.202	needles	14	14	0.119	doctors	14	16	0.189	cost	14	16	0.122
questionable	14	14	0.111	useful	14	16	0.268	human	14	34	0.076	quick	14	22	0.179
what	14	15	0.101	hope	13	14	0.172	youth	14	14	0.198	pap smear	13	14	0.132
warts	14	16	0.158	yes	13	18	0.154	immunity	14	16	0.223	relief	13	14	0.216
ok	14	18	0.102	teens	13	16	0.254	immunisation	14	16	0.227	positive	13	16	0.198
risky	14	16	0.103	painless	12	16	0.125	cost	14	18	0.239	simple	13	16	0.172
injection	14	16	0.171	really?	12	12	0.107	smart	13	14	0.136	precaution	13	14	0.246
hurt	14	16	0.235	medicine	12	16	0.171	easier	13	14	0.163	youth	13	14	0.174
test	13	14	0.131	interesting	11	11	0.112	life saving	13	14	0.279	expensive	12	12	0.096
need	12	14	0.134	none	11	32	0.089	female	13	14	0.195	uncomfortable	12	14	0.203
healthy	12	14	0.179	disease	11	12	0.123	innovative	12	12	0.141	good idea	12	14	0.194
beneficial	12	12	0.134	hopeful	11	12	0.153	beneficial	12	12	0.147	age	12	12	0.114
youth	12	15	0.165	pap	11	11	0.096	vaccination	12	16	0.135	teenager	12	16	0.235
yes	12	19	0.06	help	10	10	0.14	not sure	11	17	0.095	smart	11	12	0.166
teenager	11	14	0.186	questionable	10	10	0.065	healthy	11	12	0.181	unnecessary	11	13	0.068
female	11	12	0.15	not	10	10	0.024	expensive	11	11	0.072	immunisation	11	12	0.171
aids	11	12	0.111	innovative	10	10	0.183	teenager	11	14	0.196	when	11	12	0.157
controversial	11	12	0.135	relief	10	10	0.135	breakthrough	10	12	0.094	vital	11	12	0.203
wonderful	11	12	0.107	wonderful	10	10	0.137	hiv	10	10	0.185	how	11	12	0.096
precaution	11	14	0.147	lifesaving	10	12	0.145	vital	10	10	0.167	life saving	10	12	0.198
hiv	11	12	0.147	cost	10	10	0.108					interesting	10	11	0.031
interesting	10	10	0.081									effectiveness	10	10	0.126
untested	10	10	0.075									worthwhile	10	10	0.033
early	10	10	0.122												
good idea	10	10	0.06												
medicine	10	12	0.17												
cost	10	10	0.027												
everyone	10	12	0.244												
herpes	10	10	0.187												

Note: Deg denotes Degree; WDeg denotes weighted degree, and EVC denotes eigenvector centrality

**Fig 7:**
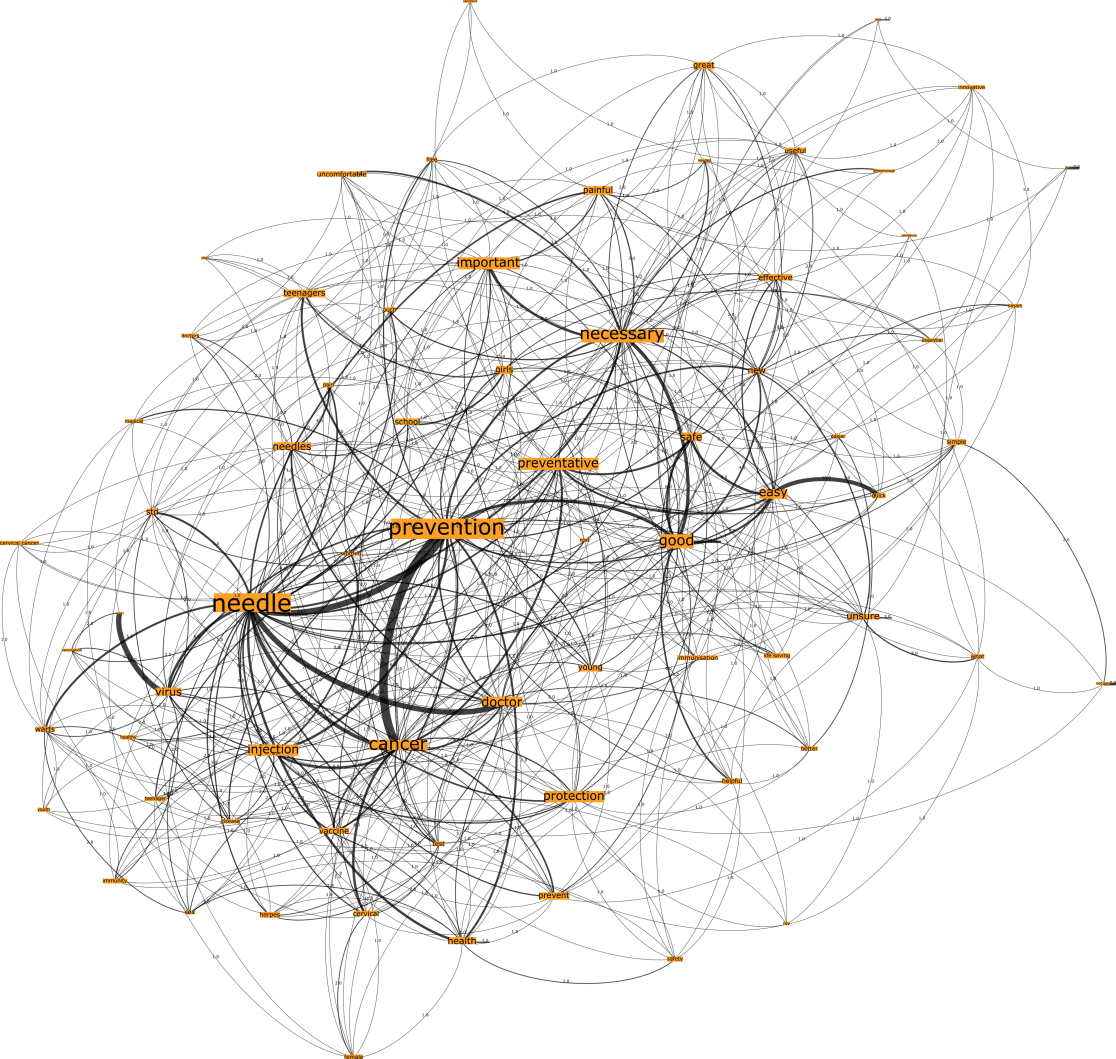
Network G. Australia HPV vaccination network visualization.

**Fig 8:**
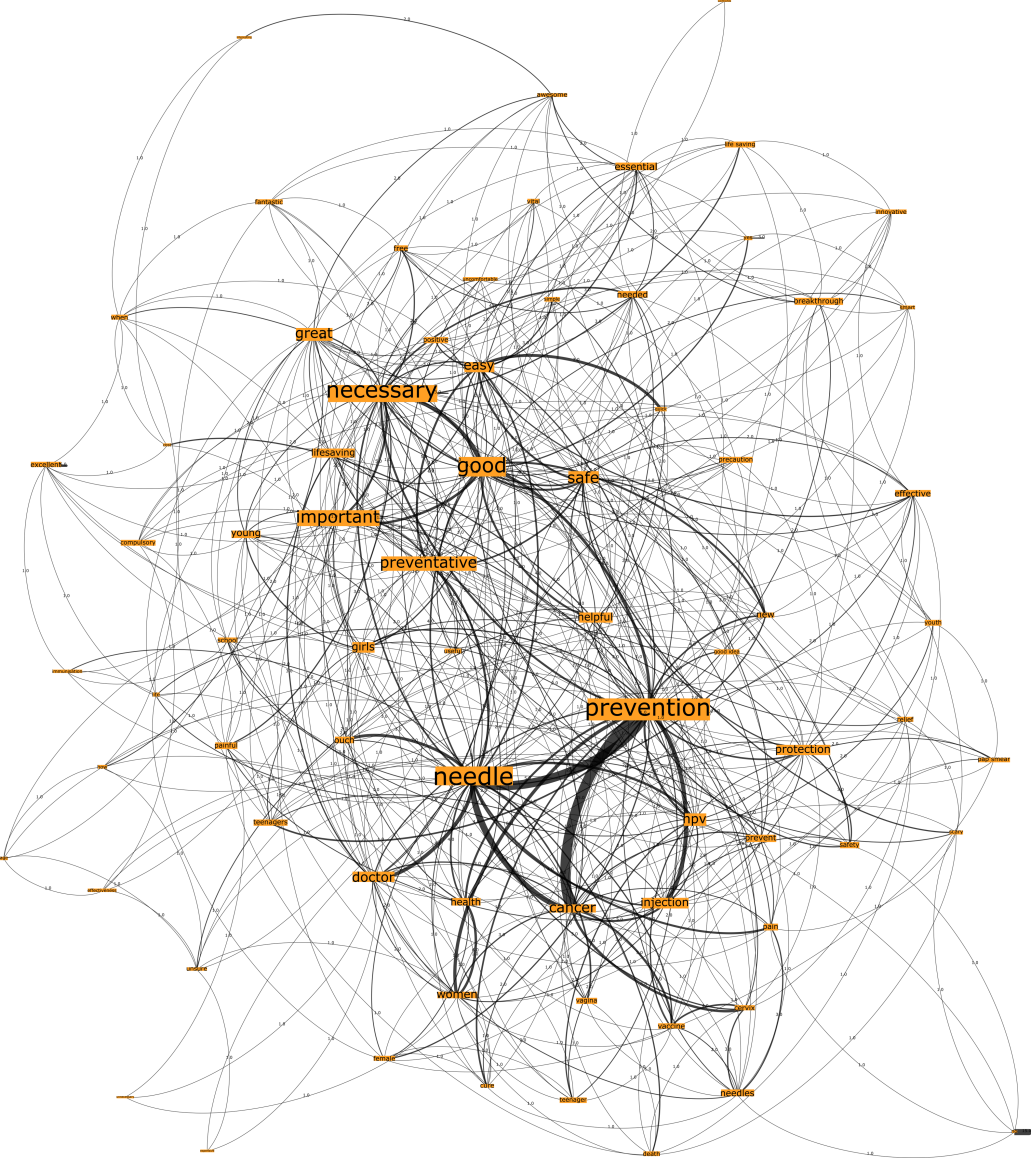
Network H. Australia cervical vaccination network visualization.

In reference to the Australian country group, Network G, which was seeded from the trigger words “HPV vaccination”, comprised 777 nodes connected by 1923 edges with an average degree of 4.95 (Fig 7). This network graph partitions into four modules, with a modularity score of 0.235. Analysis of the network shows that there is one core community related to the n-grams “prevention” and “needle”, one major community related to the n-gram “necessary” and two disparate communities. Finally, Network H, seeded by the trigger words “cervical cancer vaccination”, comprised 732 nodes connected by 1889 edges, with an average degree of 5.161 (Fig 8). This network graph partitions into three modules with a modularity score of 2.42. The communities were dispersed into two core communities and one disparate community, such that one core community related to the n-grams “prevention” and “needle”, and the other core community related to the n-gram “necessary”.

In review of Table 3, degree and eigenvector centrality measures demonstrate that the top-ranking n-grams across the Australian networks bear similarities, regarding the most embedded n-grams. Specifically, the three most embedded n-grams for the trigger word HPV vaccination were “prevention”, “needle” and “necessary”, whilst the trigger words “cervical cancer vaccination” had the n-grams “prevention”, “necessary” and “good”. It is interesting to note that the fourth ranking n-gram for HPV and cervical cancer vaccination were “good” and “necessary” respectively.

## Discussion

We structure our discussion around each of the research questions outlined at the beginning of this study. First, in spite of the differences between the ECDP across both countries, the associations regarding the trigger words HPV testing produced positive perceptual associations by female participants in the U.S. and Australian samples studied. We also found that negative connotations were raised by participants relating to the uncomfortable nature of the Pap test.

The overarching theme representing this particular form of testing was signified across both country samples by n-grams such as “uncomfortable”, “awkward” and “invasive”. This theme encapsulates the negative connotations that participants have associated with the Pap smear test, implied by the terms “discomfort”, “painful” and “awkward”. Although, the Pap smear network graphs within each country sample highlight the necessary and preventative nature of the test in identifying cervical cancer, this form of cervical cancer testing engenders negative perceptions, which may function to inhibit preventative action amongst women. Consequently, better education of health professionals is required to make the testing process and service environment less uncomfortable, which may work to increase the participation rate.

The results show that both the U.S. and Australian country samples drew links between the trigger words “HPV and cervical vaccination” and sexually transmitted infections (STIs), with the terms “sex”, “std”, “virus” and “disease” reported by the participants. Both country samples also identified a correct association between the triggers word “HPV vaccination” and the n-grams “warts”. This shows that there is knowledge in each country sample that HPV can cause genital warts and is an STI. However, both country samples identified incorrect STI associations between these trigger words (“HPV vaccination”) and the n-grams “HIV”, “herpes” and “AIDS”. Such findings might indicate that women across both country samples do not differentiate between STIs, as well as holding false assumptions about HPV testing using the same medical procedures as HIV testing (a ‘simple blood test’). Therefore, this finding illustrates potential health themes for educational public service announcements and intervention programs that encourage adoption of the HPV vaccine and preventative sexual behaviors.

Taken collectively, these findings are significant based on the explicit word choices of negative connotations toward this form of cervical cancer screening may function to inhibit women’s decision-making with regard to the adoption of preventative health behavior actions (i.e. undergoing regular Pap smear tests). Consequently, it is advisable that cervical screening education programs are designed to inform the public as to the precise details of HPV vaccination and screening schedules as well as mitigate flawed assumptions and misconceptions regarding the nature of the procedures. By addressing concerns on the part of women about timing and comfort, such action may improve HPV and Pap smear-testing goals.

Second, regarding associations surrounding HPV vaccination, we found across both country samples that women hold correct and favorable associations relating to the preventative and beneficial nature of the vaccination. For example, participants consistently referred to the n-grams “school”, “teens” and “young”. This finding signifies that participants across both country samples are aware that the vaccination is administered to pre-teen females via doctors or school immunization programs [35].

Third, in terms of broad relevance for public health research, this study reveals a number of interesting insights. In particular, the approach used in this paper allowed us to assess the diversity and variation in vocabularies used by patients to describe their perceptions and associations across two country samples. We then showed a simple method for identifying the relative importance of terms to specific trigger words.

**Fig 9:**
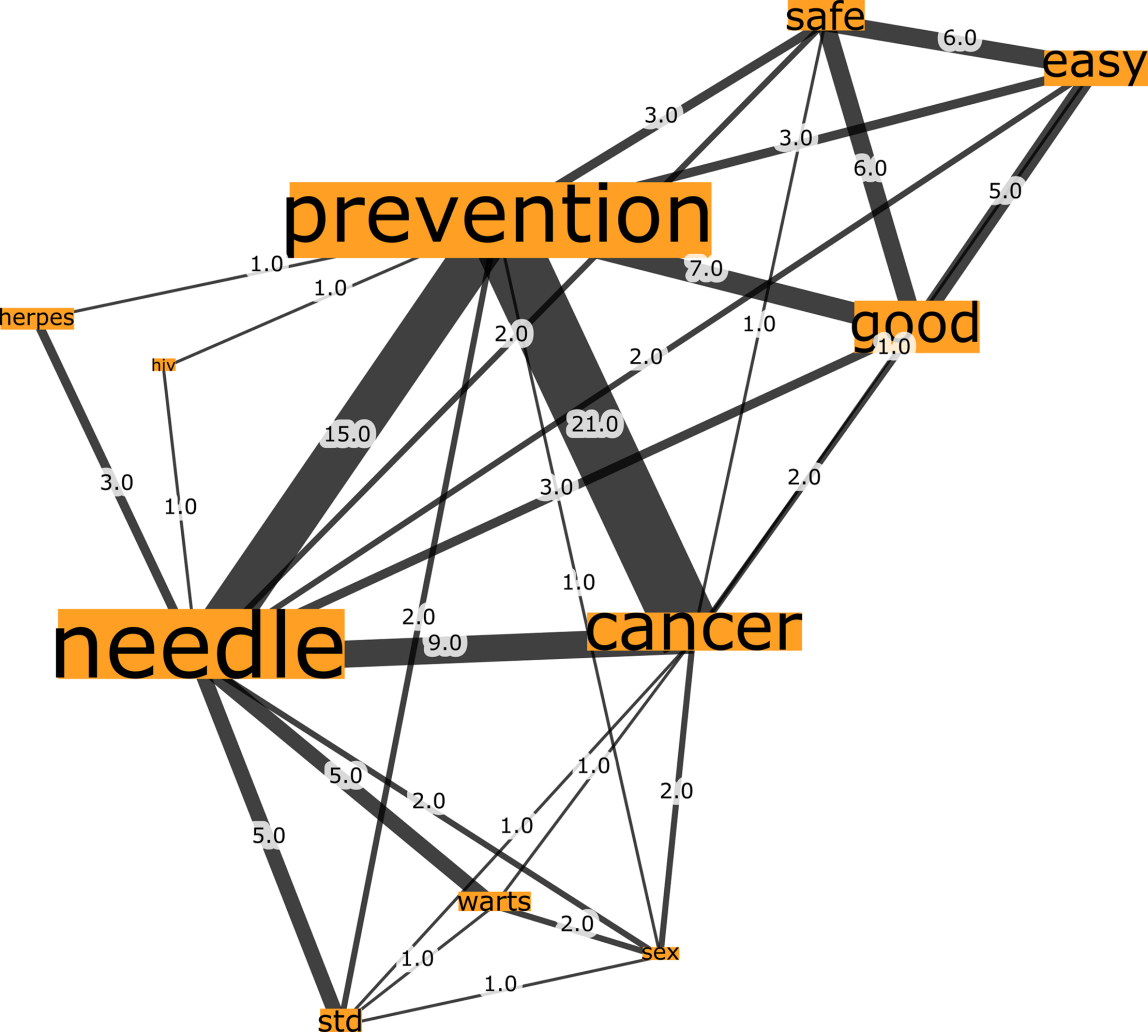
Australia HPV vaccination network graph sub-structure.

In this study, we have demonstrated the usefulness of our approach in identifying community groups and sub-structures within networked patient associations across each country sample. Further clarity can be achieved with targeted filtering of the networks to examine network sub-structures more closely. Fig 9 below, for instance, shows two prominent communities (based on node degree centrality and edge weight) extracted from the Australian HPV vaccination network (“needle”, “prevention”, “cancer” and “good”, “easy”, “safe”, respectively) and the connections shared by the nodes.

**Fig 10:**
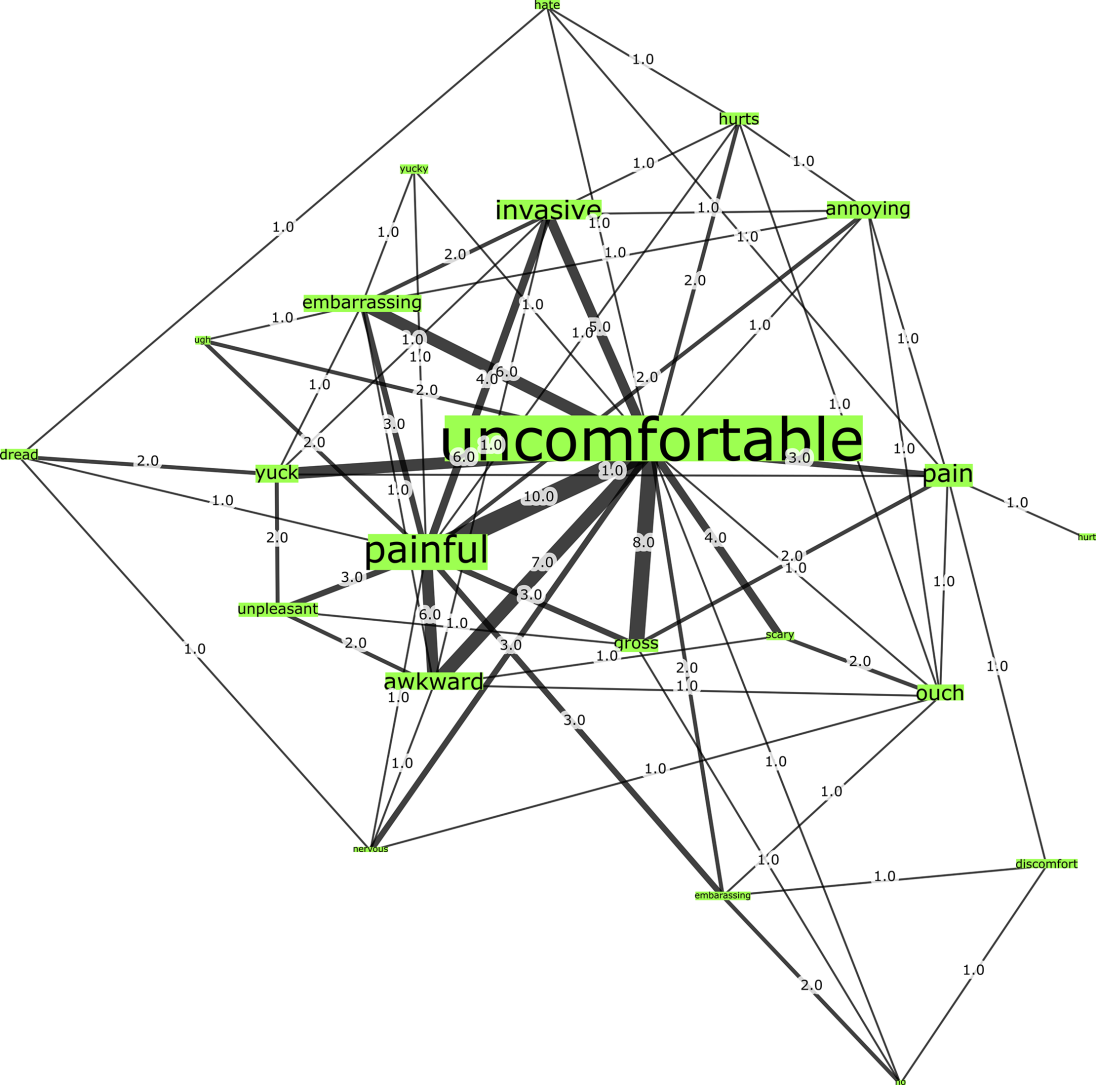
U.S. Pap smear network graph sub-structure.

We have also demonstrated how easily words pertaining to specific topics, content, or sentiment types can be compared. Fig 10 below, for instance, shows only terms possibly understood as negative filtered from the U.S. Pap smear network. This allows us to quickly compare connections between prominent nodes such as “uncomfortable” and “painful” with lower-ranked nodes such as “annoying” and “embarrassing”. Such filtering could also be used to filter negative and positive associations for comparison.

## Limitations, future research and concluding remarks

Our study has several limitations. First, the data set used in this study may not be representative of the female population aged 18+ in the targeted countries, as the algorithm for selecting participants is not disclosed and the pool of potential participants as a whole may be inherently biased. Second, the survey was conducted in a very narrow timeframe (4 days, just before the Christmas holidays), which may have introduced some bias but would also have excluded any bias from perception shifts that might have occurred in the targeted population over an extended time. Third, we based our analysis on exact string matches without any pre-processing such as stemming, lemmatization or fuzzy matching. Thus, the relevance of some concepts may be underestimated.

A number of research directions arise from this study. Using a more representative sample of the population, future research could consider deeper analysis focused on semantics [36] and also focus more on lower ranked nodes in similar networks that may reveal associations or attributions that are less prevalent but still, perhaps, important when considered across large enough groups. Along these lines, future research should explore differences between demographic groups (e.g. young vs. old, low vs. high income or education levels, metropolitan vs regional areas, cultural background, vaccinated vs non-vaccinated) as well as groups with different contextual perceptions and biases (e.g. those who consider HPV a sexually transmitted disease, and those who do not) across different country settings.

Future research will consider applying similar approaches to unstructured data (e.g. health discussions on social media [37]). This could be an important avenue for research, given the increasing use by patients of web-based tools to gather health information [38, 39]. The approach used in this study could also apply to other public health contexts (e.g. healthy eating, drug and alcohol use, and mental health). More specifically, this could include, for example, health issues themselves (e.g. risk perceptions around skin cancer) [40], other marketing related contexts (e.g. health claims) [41], health promotion (e.g. health websites and m-health applications) [42, 43, 44], and health service management contexts (e.g. value in health services) [45]. Finally, given the cross-sectional nature of the study, future research could collect and analyze word association data collected on a real-time or longitudinal basis such as through health apps [46,47].

## Supporting information

S1 File Survey. The survey presented to the probands.
(PDF)

S2 File Survey responses. The data file (.xlsx) with the survey responses and metadata.
(XLSX)
