# Supplementary material for: Examining word association networks: A cross-country comparison of women’s perceptions of HPV testing and vaccination
Source: PLoS One. 2017 Oct 5;12(10):e0185669. doi: 10.1371/journal.pone.0185669 (PMC5628849; doi:10.1371/journal.pone.0185669)
Supplement: S1 File — The survey presented to the probands. (PDF) [file pone.0185669.s001.pdf]

Survey

A 50.0% The information that you provide us will be used to value the grade of acceptance of a screening test.

Women often go to their doctor for a check for changes to the cells of the cervix that may lead to cervical cancer. This screening has halved the number of cases and death from cervical cancer.

There are now two new tests available:

The New Pap smear is better in picking up precancerous changes compared to the current smear. It will therefore detect more early changes that can be treated to prevent cervical cancer. You will still need a vaginal examination with a speculum like with the current smear.

The human papilloma virus (HPV) test is better in picking up precancerous changes compared to the current test. It will therefore detect more early changes that can be treated to prevent cervical cancer. You will still need a vaginal examination with a speculum like with the current test.

Would you rather have the HPV test or the New Pap smear when you visit your doctor?

B 50.0% The information that you provide us will be used to value the grade of acceptance of a screening test.

Women often go to their doctor for a check for changes to the cells of the cervix that may lead to cervical cancer. This screening has halved the number of cases and death from cervical cancer.

There are now two new tests available:

The human papilloma virus (HPV) test is better in picking up precancerous changes compared to the current test. It will therefore detect more early changes that can be treated to prevent cervical cancer. You will still need a vaginal examination with a speculum like with the current test.

The New Pap smear is better in picking up precancerous changes compared to the current smear. It will therefore detect more early changes that can be treated to prevent cervical cancer. You will still need a vaginal examination with a speculum like with the current smear.

Would you rather have the New Pap smear or the HPV test when you visit your doctor?

- ○ New Pap smear
- ○ HPV test

Survey

Do you consider cervical cancer to be caused by a sexually transmitted disease?

- ○ No
- ○ Yes

Survey

In the following questions you will be shown a medical term. Please enter the first three words which come to mind after reading each term. Please enter only **ONE WORD IN EACH BOX**. Try to be spontaneous as any word is relevant.

Survey

HPV vaccination

Survey

Cervical cancer vaccination

Survey

HPV (Human Papilloma Virus) test

Survey

Pap smear

Survey

New Pap smear
